# Mechanical behavior of a soft hydrogel reinforced with three-dimensional printed microfibre scaffolds

**DOI:** 10.1038/s41598-018-19502-y

**Published:** 2018-01-19

**Authors:** Miguel Castilho, Gernot Hochleitner, Wouter Wilson, Bert van Rietbergen, Paul D. Dalton, Jürgen Groll, Jos Malda, Keita Ito

**Affiliations:** 1 Department of Orthopaedics, University Medical Center Utrecht, Utrecht University, GA Utrecht, 3508 The Netherlands; 2 Orthopaedic Biomechanics, Department of Biomedical Engineering, Eindhoven University of Technology, Eindhoven, MB 5600 The Netherlands; 3 Regenerative Medicine Center Utrecht, CT Utrecht, CT 3584 The Netherlands; 4 Department of Functional Materials in Medicine and Dentistry, University of Würzburg, Würzburg, 97070 Germany

## Abstract

Reinforcing hydrogels with micro-fibre scaffolds obtained by a Melt-Electrospinning Writing (MEW) process has demonstrated great promise for developing tissue engineered (TE) constructs with mechanical properties compatible to native tissues. However, the mechanical performance and reinforcement mechanism of the micro-fibre reinforced hydrogels is not yet fully understood. In this study, FE models, implementing material properties measured experimentally, were used to explore the reinforcement mechanism of fibre-hydrogel composites. First, a continuum FE model based on idealized scaffold geometry was used to capture reinforcement effects related to the suppression of lateral gel expansion by the scaffold, while a second micro-FE model based on micro-CT images of the real construct geometry during compaction captured the effects of load transfer through the scaffold interconnections. Results demonstrate that the reinforcement mechanism at higher scaffold volume fractions was dominated by the load carrying-ability of the fibre scaffold interconnections, which was much higher than expected based on testing scaffolds alone because the hydrogel provides resistance against buckling of the scaffold. We propose that the theoretical understanding presented in this work will assist the design of more effective composite constructs with potential applications in a wide range of TE conditions.

## Introduction

Soft tissues, *e*.*g*. articular cartilage or cardiac muscle, are complex composite materials consisting of collagen fibrils reinforcing a cell-containing proteoglycan gel-like matrix^1,2^. To mimic such natural composite structures, recent studies have highlighted the potential of developing composite constructs composed of hydrogels and fibrous materials^3–5^. Hydrogels, with their highly hydrated polymer network, can provide the cells an environment that further resembles the natural extracellular matrix (ECM)^6^, whilst the reinforcing fibres can provide the necessary mechanical properties, close to those of biological tissues^7^. Preparation of such composite materials usually involves a two-step procedure, *i*.*e*. fabrication of the fibre reinforcing scaffold and then crosslinking a gel inside these fibre scaffolds^3,8^. Electrospinning and wet spinning are commonly used techniques for fibre formation due to their relative simplicity and capability to produce sub-micron diameter fibres^9,10^. However, their difficulty in manufacturing controlled architectures and three-dimensional (3D) complex geometries compromises cell infiltration^11^ and seriously limits hydrogel mechanical reinforcement^12^. Recently, it was demonstrated that with a direct melt electrospinning writing (MEW) process, or melt electrowriting as recently designated^13^, micro-fibre scaffolds could be fabricated with well-controlled 3D architectures^14,15^. This electrohydrodynamic process forms fibres from polymer melts to deposit highly defined networks, with fibre diameters within the range of 800 nm^16^ to 150 µm^17^.

The inclusion of melt-electrospun scaffolds within hydrogels can be used to improve the mechanical properties of hydrogels and simultaneously modulate their mechanical response. Visser *et al*. demonstrated that the combination of polycaprolactone (PCL) MEW scaffold with a soft gelatine-methacryloyl (GelMA) hydrogel synergistically enhance the compressive properties of the composite hydrogel constructs, while still exhibiting an excellent biological performance^18^. This synergistic effect was assumed to be due to the fibres being pulled in tension when the construct was loaded in compression, *i*.*e*. a Poisson’s effect. This assumption was supported by a relatively simple mathematical model that considered the hydrogel and fibre scaffolds as composed of an idealized geometry and linear elastic materials. Nevertheless, it over-predicted the final stiffness of the composite constructs by one order of magnitude. Further studies using GelMA and GelMA/hyaluronic acid-methacrylamide gels reinforced with similar MEW scaffolds corroborate the same theory, although a different reinforcement level was observed as compared to Visser *et al*.^19^. Despite the great potential of the composite constructs, the reinforcing effect of the MEW scaffold is thus not fully understood. The absence of an adequate framework to explain the observed reinforcement mechanism is limiting the optimisation of these novel designs and is hampering their application. The use of computational methods, that could simulate the mechanical behaviour of these composite constructs, can bring valuable insights into the process that governs the reinforcement mechanism, although, the accuracy of these methods is dependent on the precise definition of the material properties, model geometry and interaction between the composite constituents. Also, the difference in mechanical behaviour between the composite components, *i*.*e*. soft hydrogels reinforced with stiffer microfibres, is known to lead to non-linear phenomena, e.g. fibre buckling and structural instabilities, that impact on the reinforcement mechanism^20^. Therefore, the aim of the present study was to elucidate the mechanics of the reinforced hydrogel composite through FE simulations. Two different FE models were used. The first is a continuum model representing a schematized hydrogel and scaffold geometry (either individually or combined), to investigate the load carrying capacity of hydrogel and scaffold alone, and the reinforcement mechanism due to the scaffold restricting hydrogel movement in composite constructs. The second is a micro-FE model of the scaffold without and with hydrogel, to investigate the actual (buckled) geometry of the scaffold at different states of compression and the load transfer directly through the interconnections of the scaffold at these stages. By combing the simulation results with the experimental results, material parameters of the composite constituents are derived and the reinforcement mechanism of the fibres can be explained in detail.

## Results

### Construct fabrication and mechanical characterization

**Figure 1:**
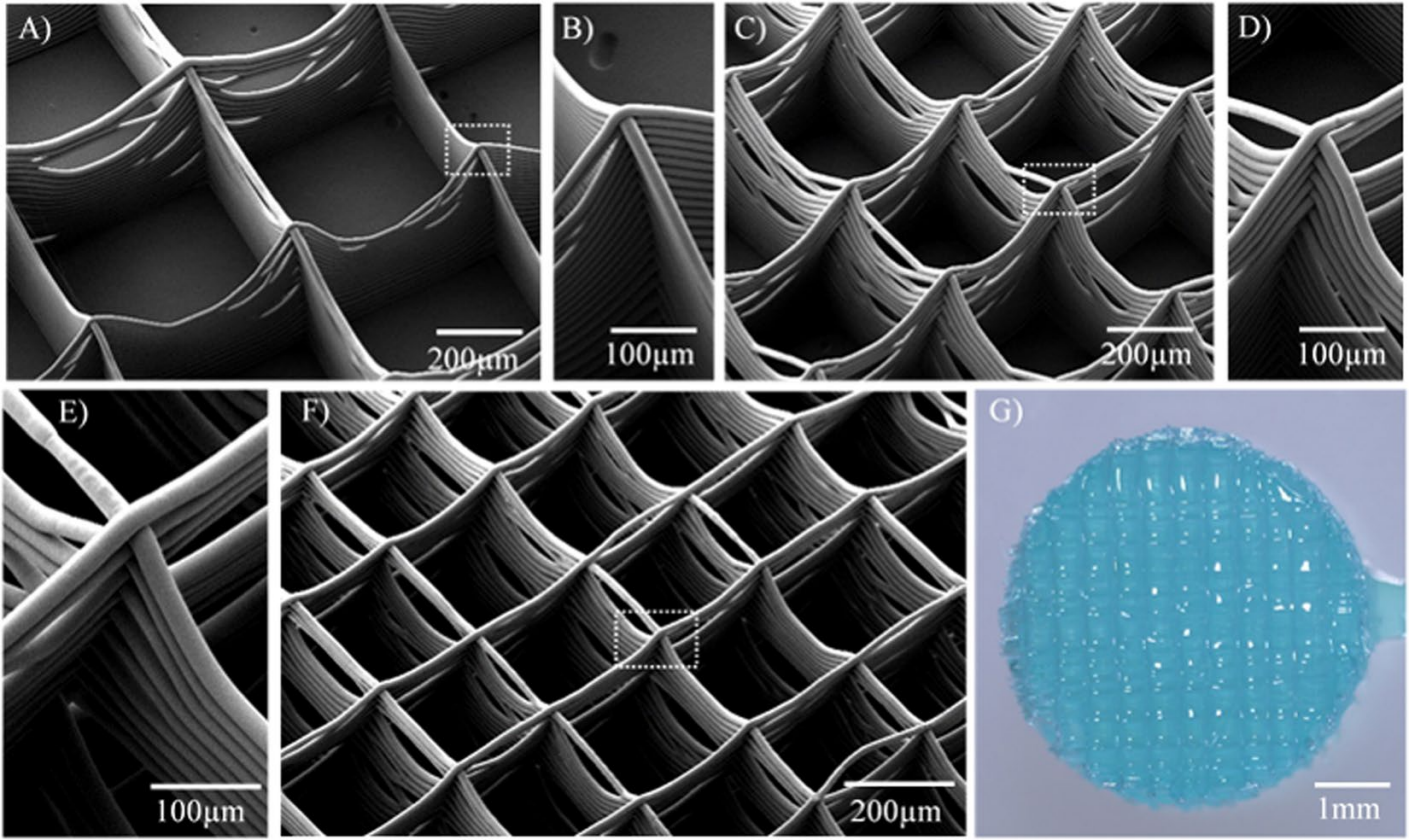
Scanning electron microscopy images of MEW printed fibre scaffolds with details of the buildup fibres at the fibre interconnections. Scaffold with a filament spacing of (**A**) (**B**) 800 µm, (**C**) (**D**) 400 µm, and (**E**) (**F**) 200 µm. (**G**) Stereomicroscopic image of the GelMA hydrogel reinforced with a fibre scaffold with a filament spacing of 200 µm.

Figure 1 shows the microstructure of fabricated electrospun scaffolds with different fibre volume fractions. While the accurate fibre placement onto the collector enabled the formation of controlled squared fibre arrangements in two-dimensions (2D) (Fig. 1A–F), some fibre sagging occurred towards the middle of the fibre walls, which was more evident for scaffolds with larger fibre spacings (Fig. 1A–F). This is due to the fact that each scaffold was generated with a single fibre filament drawn continuously, which generated multiple crossing fibres at the square edges. Moreover, the efficient combination of the micro-fibre scaffold within the GelMA hydrogel was confirmed (Fig. 1G). As the fibres were written with melt-extrusion, fibres were mostly fused together, forming a membrane-like wall with occasional openings. Similarly, at crossing points the fibers were fused forming a column-like structure.

A thorough mechanical evaluation of the composite constructs was further performed to evaluate the material properties required for the FE models. Tensile tests on single fibre filaments (Supplementary Fig. S1) revealed an elastic fibre modulus of 363 ± 21.2 MPa, which is within the range previously reported for melt extruded PCL filaments^21,22^. The compressive properties of the fibre scaffolds alone, GelMA hydrogel alone and respective composites are summarised in Table 1. A compression modulus of up to E = 387 ± 34.6 kPa at v_f_ = 7.0% ± 0.3% was found for fibre reinforcement constructs, while non-reinforced GelMA constructs exhibited a compressive modulus of E = 8.2 ± 0.9 kPa and PCL scaffolds alone E = 14.1 ± 1.9 kPa at same of v_f_ = 7.0% ± 0.3%.

**Table 1:** Measured volume fraction, V_f,_, of the reinforcing fibre scaffold, and compressive modulus, E, of the composite construct and its hydrogel matrix and fibre scaffold phase alone. Values are mean ± SD.

	Hydrogel	Composite construct			
Fibre filament spacing, A in mm	—	0.8	0.6	0.4	0.2
Fibre volume fraction, V_f_ in %	—	3 ± 0.2	4 ± 0.1	6 ± 0.2	7 ± 0.3
E hydrogel matrix in kPa	8.2 ± 0.9				
E fibre scaffold phase in kPa		4.2 ± 0.5	4.2 ± 0.8	8.4 ± 1.3	14.1 ± 1.9
E composite in kPa	—	81.4 ± 10.3	122.2 ± 16.21	207.43 ± 40.1	387.5 ± 34.6

### Finite element analysis

**Figure 2:**
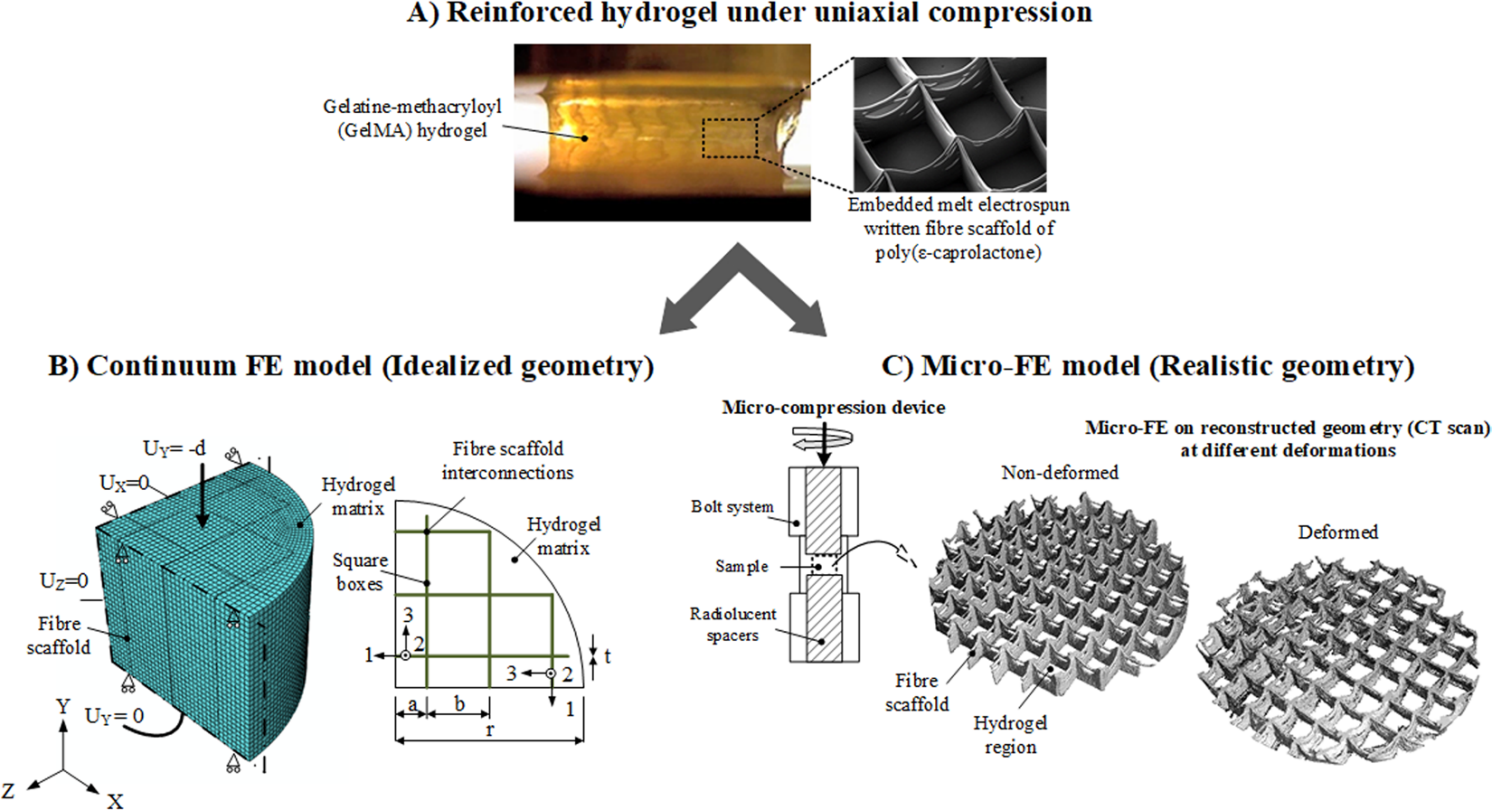
Overview of modeling workflow for both FE models proposed under uniaxial compression. (**A**) Experimental analysis of fibre reinforced hydrogels mechanical behavior under uniaxial compression to access material properties and validate numerical predictions. (**B**) Continuum FE model of idealized composite architecture. Only a quarter of the composite construct was modelled with the reinforcing box scaffolds idealized as thin laminas (defined in a local coordinate system) embedded in a hydrogel matrix. At the fibre scaffold interconnections a truss element was added with its longitudinal axis in the global y-direction. (**C**) Micro-FE model of the real composite architecture at different deformation levels. Real architecture was accessed at increasing levels of compactaction with the use of a micro-compression device, as schematically represented, and a high-resolution CT scanner.

The workflow of the developed FE modelling methodologies is briefly summarized in Fig. 2. For the continuum FE model an idealized model of the composite construct geometry was first generated and the material properties of each constituent, i.e., hydrogel and fibre scaffold, were determined by fitting a FE model of each constituent to the experimental tests performed on isolated materials. Subsequently, the determined constitutive parameters were implement in the FE model of the composite constructs for various fibre volume fractions which was then compared with the experimental data. For the micro-FE model, the actual construct geometries at different compressive strain levels were used to build the model. The constitutive parameters used in the micro-FE were determined using the methodology detailed in the methods section.

### Continuum FE model

**Figure 3:**
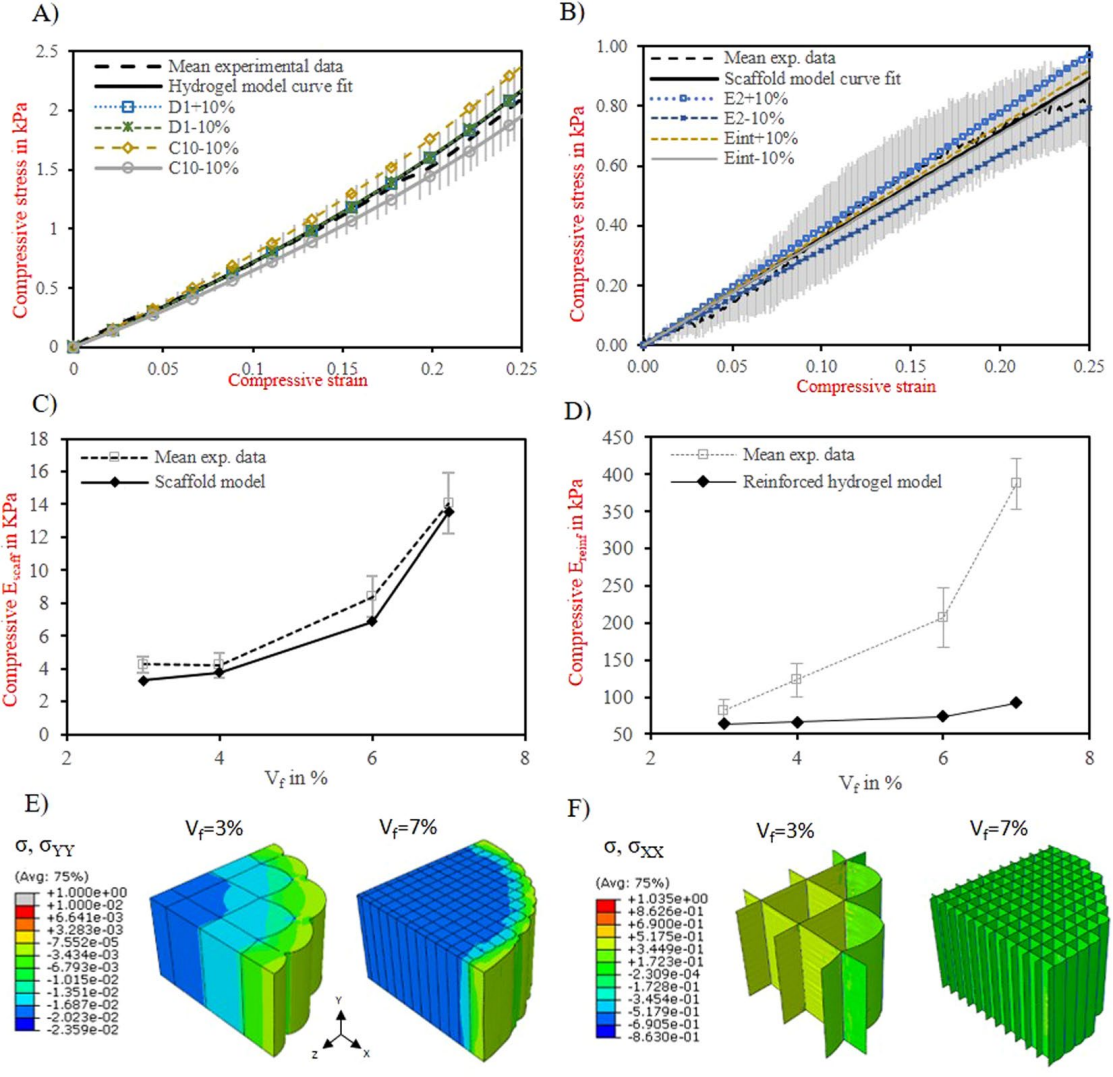
Continuum FE model results under uniaxial compression. (**A**) Fit between mean experimental stress-strain curves of hydrogel matrix (dashed black line) and hydrogel FE prediction (solid black line). Sensitivity analysis of the fitted hydrogel material parameters: D1+10% (dotted blue line); D1–10% (dashed green line, C10+10% (dashed orange line), C10–10% and comparison with experimental stress-strain curves. (**B**) Fit between mean experimental stress-strain curves of the reinforcing scaffold with vf 3% (dashed black line) and respective scaffold FE prediction (solid black line). Sensitivity analysis of the fitted fibre scaffold material parameters: E_2_ + 10% (dotted blue line); E_2_–10% (dashed blue line), E_int_ + 10% (dashed orange line) and E_int_ −10% (solid grey line). Error bars represent the experimental standard deviation of a given stress value (n = 5). (**C**) Comparison of the predicted and experimental stiffnessess for the different fibre scaffolds alone (E_scaff_) as a function of their fibre volume fraction. (**D**) Comparison of the predicted and experimental stiffnessess for the reinforced constructs (E_reinf_) under compression loading, as a function of the fibre volume fraction, vf. Fibre diameter considered Ø = 20 µm. Distribution of the (**E**) transversal and (**F**) longitudinal stress for the reinforced construct and fibre scaffold, respectively, for the volume fraction of 3 and 7%. Stress is in MPa.

The two parameters of the Neo-Hookean constitutive model for the hydrogel were obtained by fitting the unreinforced hydrogel FE model to the respective experimental results. Table 2 summarizes the determined coefficients and good agreement between the model results and the experimental stress-strain data was observed (Fig. 3A). A sensitivity analysis revealed that the material behavior showed a strong dependency on the estimated shear modulus (C_10_ parameter). However, even changing the C_10_ parameter by 10%, the behavior fell within the spread of the experimental data.

**Table 2:** Determined constitutive material parameters for the hydrogel matrix and for the fibre scaffold used for the numerical analysis. Membrane properties were defined according to the local coordinate system (see Fig. 2B).

Component	Material	Material properties	Constitutive material properties
*Continuum FE*			
Hydrogel matrix	GelMA	Neo Hookean hyperelastic	C_10_ = 0.00135 MPa; D_1_ = 30 MPa^-1^
Fibre scaffold	PCL	*Square boxes*	
		Linear elastic orthotropic membranes	E_1_ = 182 MPa; E_2_ = 0.06 MPa; υ_12_ = 0.43; G_12_ = 0.88 MPa; G_13_ = 0.91 MPa; G_23_ = 1E10^–7^ MPa
		*Interconnections*	E _int_ = 3 MPa
		Linear elastic	(Loaded cross section area 1.35E10^–4^ mm^2^)
*Micro FE*			
Hydrogel matrix	GelMa	Linear elastic	E_gel_ = 0.0082 MPa; υ_gel_ = 0.49
Fibre scaffold	PCL		E_fib scaff_ = 0.057 MPa; υ_fib scaff_ = 0.3

The fibre scaffolds were idealized as unidirectional thin laminas organized in a box structure, with perfectly parallel micro-fibres that could barely withstand stresses in any direction other than that of the micro-fibres. The interconnections, at box vertices, were idealized as columns, due to the multiple inter-weaving of crossing-fibres (Fig. 2B). Table 2 shows the determined material properties accessed by the tensile tests on single fibres and by fitting results calculated from FE analyses representing an uniaxial compression simulation of a fibre scaffold alone with V_f_ = 3% to the respective experimental data. Sensitivity analysis evidenced a strong dependency of the scaffold stiffness on the lamina transversal modulus, E_2_, and on the fibre interconnection stiffness, E_int_ (Fig. 3B). With the determined material properties, the FE model of the fibre scaffold alone represented well the experimentally measured stiffness for different fibre-volume fraction (Fig. 3C)). Yet, it must be noticed that the lamina longitudinal modulus (182 MPa) was assumed constant and evaluated from single fibre stress-strain curves at 1–3% strain levels (see Fig. S1), whereas experimentally fibres were observed to stretch to approximately 10% longitudinally when subjected to a compressive axial strain of 30%. For this reason, the analyses were repeated using a longitudinal modulus determined at a strain level of 10% (80 MPa) and the predicted composite modulus was compared with experimental results. The results demonstrated that the continuum FE model with the new longitudinal modulus implemented still significantly underestimated the modulus of the higher fibre volume fraction composites, as previously observed for a lamina longitudinal modulus of 182 MPa. This indicates that the conclusions would not change if we took the lamina longitudinal modulus at 10% strain.

To further evaluate the validity of the continuum FE model for the composite constructs, we have first assessed the ability of the model to capture the hydrogel confinement mechanism. The model predicts an axial area expansion of approximately 16% and 5% in V_f_ = 3% and V_f_ = 7% composites, respectively. This corresponded well to the area expansion measured during the experiment, 15–21% in V_f_ = 3% composites and 3–7% in V_f_ = 7% composites. Results are also similar to our previous experiments on similar composites where axial area in V_f_ = 3% composites was observed to expand between 17–23%^18^.

Moreover, a good agreement was observed between the measured and predicted stiffnessess for the constructs with a low fibre volume fraction (Fig. 3D–F). However, the continuum FE model significantly underestimated the modulus of the higher fibre volume fraction composites, being approximately four times lower than the experimental modulus. This could suggest that the scaffold moduli that were determined by fitting the FE model with the experimental results for the scaffold alone, are not representative for those of the scaffold in the composite constructs, e.g. because the scaffold deformation in the presence of the gel is very different from that of the scaffold alone. To further investigate this, the micro-FE model was used.

### Micro-FE model

**Figure 4:**
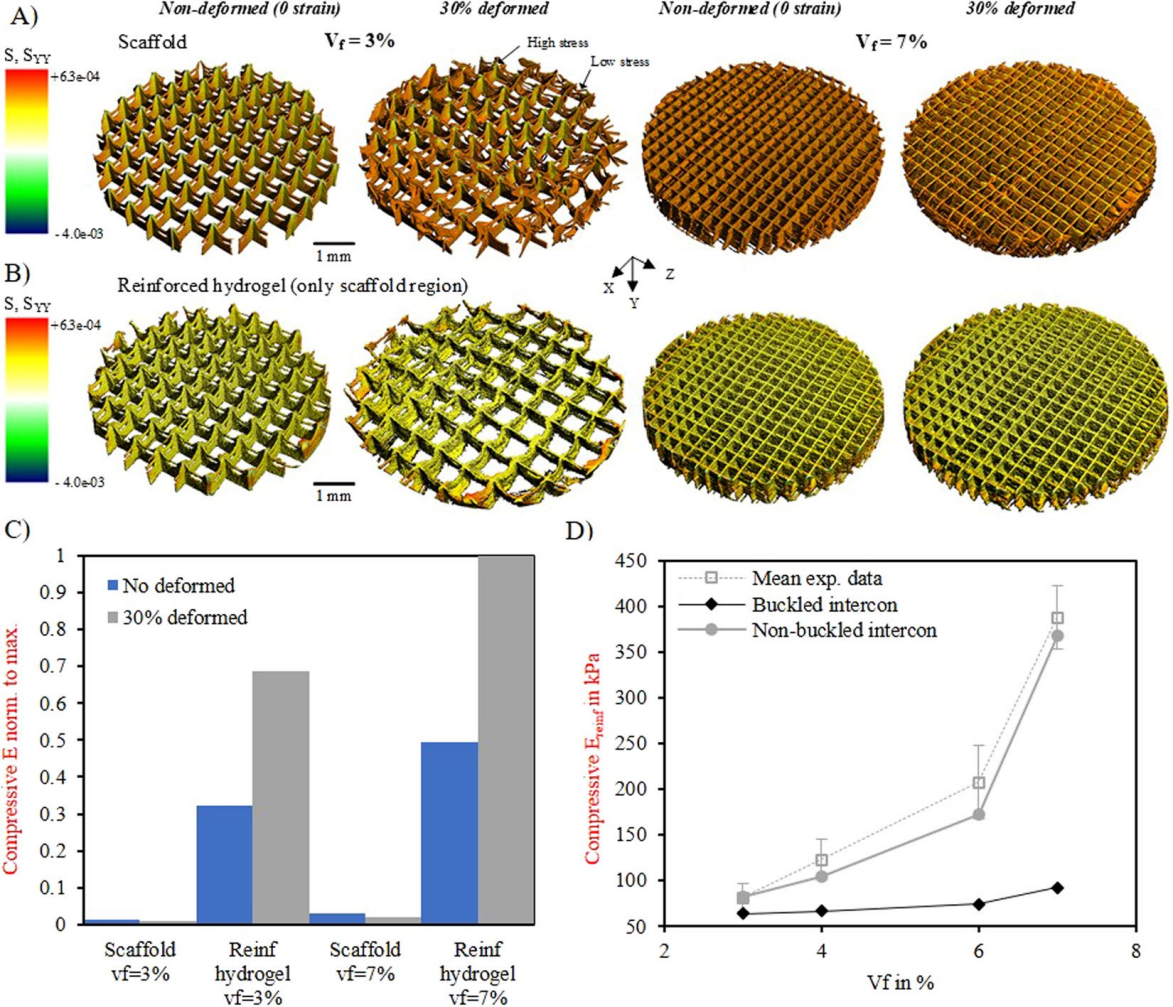
Micro-FE model results. (**A**) Comparison of loaded regions between the A) fibre scaffold and the (**B**) reinforced hydrogel with a volume fraction of 3% and 7%. Loaded regions are represented as compression stress and were determined by micro-FE analysis on non-deformed and deformed real geometries. Stress is in MPa. (**C**) Stiffness predicted from micro-FE analysis. Stiffness was relativized by dividing each value by the maximum value. (**D**) Comparison of the predicted and experimental stiffnessess for the reinforced constructs (E_reinf_) with buckled and non-buckled interconnections stiffness using the continuum FE model. Interconnections material properties were re-fitted to the composite construct with a v_f_ = 3%, where no buckling was observed.

The micro-FE model was created directly from 3D micro CT reconstructed geometries of both fibre scaffold alone and reinforced gel at compressive strain levels of 0, 15, 30 and 45% (data not shown for 45% strain). Preparation of the reconstructed geometries involved the use of a micro-compression device to apply and maintain strain^23^ (Fig. 2C), and the use of a micro-CT to image the constructs in the given strain configurations. Subsequently, reconstructed images were converted to micro-FE models, using a voxel conversion procedure^24^ and the construct stiffness in the deformed configuration was determined from a micro-FE analysis. Table 2 summarizes the material properties determined by using the fitting procedure summarized in the Methods section, Micro- FE analysis. While evaluating the deformed geometries and compressive stress distribution of scaffolds alone and scaffolds combined with hydrogel, a clear difference was found (Fig. 4 A,B). In the non-deformed configurations, the maximum stress level was predominantly localized at the top crossing fibre regions for the scaffold alone and more uniformly distributed through the scaffold walls and interconnections for the scaffold combined with hydrogel. The stress distribution within the scaffold alone for the deformed conditions (*i*.*e*. 30% strain) showed a totally different behavior compared to the non-deformed condition. Stress was clearly localized on the few unbuckled interconnections, while the scaffold walls remained more or less unloaded. In contrast, in the scaffold combined with hydrogel the fibre walls and especially the interconnections remained straight even at larger strains. Moreover, differences in deformation between scaffold alone and scaffold embedded in the hydrogel matrix were consistent with the measured stress-strain response (Fig. S2). For the scaffolds alone stress is approximately linear up to 15% falling gradually thereafter, while for composite constructs stress is reasonably linear up until larger strains, ≈30 and 45%.

When calculating the stiffness for the deformed scaffolds shapes based on the micro-FE analyses (Fig. 4C), it can be seen that the scaffolds alone have a very low stiffness but when combined with hydrogel their stiffness increases up to 47 fold, caused mostly by prevention of scaffold structural buckling. Also, we observed that the stiffnessess at 30% strain were higher than at 0% strain (*i*.*e*. non-deformed), and that the reinforced constructs with higher volume fraction had an approximately 1.5 fold increase in stiffness. This suggested a strong dependence of the interconnections’ load transfer on the reinforcing mechanism. Therefore, the stiffness of reinforced constructs predicted using the continuum FE model and material properties of the fibre scaffold interconnections assessed in a non-buckled versus buckled status for various fibre-fractions were compared (Fig. 4D). The non-buckled interconnections stiffness (E_Non buckled intercom_ = 24 MPa) were determined from the composite scaffolds (V_f_ = 3%) where no significant buckling of the intersections was observed (Figs 4B and S2). This value is still considerable less than the material properties for the PCL fibres (363 ± 21.2 MPa), which likely is due to the fact that the melted contact area between stacked fibres can be much smaller than the cross-sectional area of the fibres (Fig. 1E) and because the interconnections do not form exactly along straight lines. It is, however, much higher than the interconnection modulus determined earlier for the scaffold alone (3 MPa, Table 2), that represents that of the buckled interconnections. For high fibre volume fractions the increasing influence of the non-buckled interconnections stiffness can be observed, as the simulated stiffness closely followed the experimental results.

## Discussion

While previous works have accessed the compressive reinforcement mechanism of hydrogel/scaffold composites related to the stretching of the fibres due to lateral expansion, in this work we are the first to consider the reinforcement mechanism due to the load transfer through the scaffold interconnections and the effect of buckling thereon. Our simulations were carried out for material properties assessed on both composite constituents independently, using experimental results, so that the predictive nature of the models as a function of fibre fraction could be validated. The important outcome of this work is that the reinforcement mechanism of the composite constructs was found to be governed by two different mechanisms. The first contribution is created by the fibres being pulled in tension by the lateral expansion of the hydrogel (Fig. 3E,F)). This mechanism dominates the reinforcement of composite constructs at lower fibre volume fractions where the number of fibre intersections is reduced, and was demonstrated by the continuum FE model. The second contribution comes from the interconnections. These interconnections are structurally more resistant than fibre walls, since they result from multiple interlocked fibres. However, the load carrying capacity of these interconnections is hardly recognized when testing scaffolds alone, due to extensive buckling of the connections. In the composite constructs, on the other hand, the surrounding hydrogel material provides lateral support and stability for the interconnections, preventing the buckling collapse observed for the scaffolds alone, and transferring the compressive load into the reinforcing fibres oriented longitudinally. The fibre interconnections behavior was observed to dominate the reinforcing mechanism for higher fibre volume fractions where they are more plentiful. It is noteworthy that buckling deformation was examined indirectly using a micro-compression device that applied and maintained strain. Each individual micro-FE model was linear, but generated for each non-linearly deformed configuration, and only used to assess the stiffness in that deformed state. Further modelling efforts might include the observed material non-linearities, buckling effects and micro-fibre scaffold detailed geometries in a single continuum non-linear model, although such methods are computationally expensive and time-consuming.

Recent work suggested that the reinforcement mechanism was attributed to both fibre-stretching and to the high interfacial shear stresses between the hydrogel-fibres, which prevent hydrogel from flowing out the fibre scaffold^18^. However, it was not supported by methodological calculations and further studies are required to elucidate such an assumption. Our current continuum FE model has the potential to be extended to include the rate-dependent features of the composite material and, therefore, capture such hydrogel flow. This framework can also enable to better evaluate the mechanical environment that cells will experience, which could play an important role in mediating the cell differentiation process in these novel composites^25^. Finally, we believe that future modelling approaches should explore the reinforcement mechanism under other different loading conditions, such as shear and tension, since they play an important role in human body tissues mechanical behavior.

### Conclusion

In the present study, we demonstrated that the reinforcement mechanism of the composite constructs is governed by two different mechanisms. The first is the pulling of the fibres in tension by the lateral expansion of the hydrogel. This mechanism dominates the reinforcement of composite constructs with lower fibre volume fraction. While the second contribution comes from load transfer through the fibre cross-section interconnections. The latter is not observed in scaffolds alone, due to buckling of the interconnections, but in the case of hydrogel composites, where the gel avoids the buckling this reinforcing mechanism dominates above a certain fibre volume fraction. Our results also provide new fundamental insights into the real structural deformation of these novel reinforced hydrogels during axial compression that can be used for assisting the design of more effective composite constructs.

## Methods

### Materials, fabrication and characterization

For this study, a special custom-built MEW device was developed. Air pressure regulator (FESTO, Berkheim, Germany) was used to feed the polymer melt through a 23 G spinneret charged by a high voltage HV source (LNC 10000–5 pos, Heinzinger Electronic GmbH, Rosenheim, Germany). In order to enable an additive manufacturing process, a planar movable aluminum collector plate was mounted on top of 2 orthogonal placed axes (XSlide, Velmex, New York, USA) and controlled via an advanced 2-axis stepper motor controller (PMX-2EX-SA, ARCUS Technology- Inc., USA). For a suitable temperature control of the polymer melt an electrical heating system (TR 400, HKEtec, Germany) was used. To assure stable manufacturing conditions the processing parameters were adjusted in accordance with a previous study^18^. Accordingly, the used acceleration voltage was set to 5.5 kV at a spinning gap of 3.3 mm (E = 1.3 kV/mm), a feeding pressure of 3.0 bar and a heating temperature of 90 °C was sufficient for processing GMP-PCL (PURASORB PC 12, Corbion Inc., Gorinchem, Netherlands) fibres with a desired diameter of approximately 20 µm. The collector velocity was set at 10 mm/s. Single fibres were firstly collected on a microscopic glass slide for single filament tests. Box structured meshes (5 × 5 cm^2^) were fabricated by alternating layer deposition orthogonally, with a filament spacing of 200, 400, 600 and 800 µm. The final height of every scaffolds was set to 1000 µm. The procedure for the preparation of GelMA-reinforced hydrogel constructs was described in detail in a previous study^18^. MEW printed single fibres and scaffolds were imaged using a scanning electron microscopy (SEM) (Zeiss CB 340, Carl Zeiss Microscopy GmbH, Götttingen, Germany), at an acceleration voltage of 1–2.0 kV and a working distance of 3–7 mm. Finally, the porosity of the scaffolds (Φ) was measured gravimetrically and the PCL solid volume fraction, v_f_, was determined according to, 1 − Φ.

To access the mechanical properties of the electrospun scaffolds, single-filament tensile measurements (n = 5) were performed using a micromechanical tester (BOSE ElectroForce 5500, BOSE Corp., Framingham, USA) with a 250 g load cell. Fibres with a length of 3.0 mm were fixed on a cardboard sheet as shown in Supplementary Fig. S1. Tests were conducted at a rate of 1.0 mm/min, according to ASTM D3373 standard for single-filament testing^26^. The fibre elastic modulus was calculated from engineered stress-strains curve at 1–3% strain. Uniaxial unconfined compression tests were performed on GelMA, PCL scaffolds and composite constructs, using a Dynamic Mechanical Analyzer (DMA 2980, TA instruments, New Castle, DE, USA). Cylindrical geometries with a diameter of 5 mm and a height of 2 mm were used and tests were carried out at a strain rate of 25% min^−1^ at room temperature, according to a previous study^18^. A minimum of 5 samples for each type was tested. The compressive modulus was calculated from the engineering stress-strain curves halfway the total strain range used at 12–17% strain^18^, using the unloaded cylindrical cross-sectional area for the hydrogel alone and composite constructs, and the original cross-sectional area for the fibre scaffolds. The lateral deformation of both composite constituents was captured during the compression tests with a high resolution digital microscope (Dino-Lite AM7515MT8A, Naarden The Netherlands) and analysed with Dino-Lite software. Images from the top of these constructs were also taken with the digital microscope and the help of two glass slides, at the compressive strain levels of 0 and 30%.

### Continuum FE model

#### Geometry and modelling assumptions

Fibre scaffold was idealized as unidirectional lamina (Fig. 2B). The reinforcement was simulated by placing a fibre scaffold inside of a cylindrical hydrogel matrix. An embedded to model the reinforcement mechanism. In this approaelement feature available in the Abaqus package was usedch, an embedded equation between the two meshes was created, which constrained the translation degrees of freedom of the fibre scaffold nodes (embedded part) to those of the hydrogel (host part). The elements of the hydrogel were defined by linear brick elements. The laminas were modelled by membrane elements representing the thin plate-like structure of the scaffold. At the interconnections, however, an additional truss element was added with its longitudinal axis in the global y-direction. This truss element represents the additional stiffness at the intersection due to the crossing of the fibres (Fig. 2B). Due to symmetry, only a quarter of the composite construct geometry was considered. Similar to the experimental uniaxial compression tests described above, a vertical displacement, d, was prescribed on the top of the gel surface, such that the experimental strain rate of 25% min^−1^ was simulated (Fig. 2B). The bottom gel surface was constrained in the compression direction and free to deform horizontally. Symmetric boundary conditions were applied to the hydrogel inner faces. Geometrically nonlinear analyses were performed. The details concerning the mesh and the geometry dimensions are given in Supplementary Table S1. FE models of pure GelMA hydrogel and of each individual scaffold were also built with the same characteristics to determine the unknown material properties for each composite constituent.

#### Constitutive models

A hyperelastic material model was chosen to characterize stress-strain behavior of the GelMA hydrogel matrix. Here, we assumed that the hydrogel matrix is homogeneous and isotropic, and therefore a simple Strain Energy Density (SED) function U, known as a Neo-Hookean function, that requires only two material parameters to be determined, the C_10_ and D_1_, was examined. The Neo-Hookean SED can be defined as,

1 $$U(\overline{{I}_{1}},\,J)={C}_{10}(\overline{{I}_{1}}-3)+\,\frac{1}{{D}_{1}}{(J-1)}^{2}$$

where $\overline{{I}_{1}}$ is the first strain invariant of the left Cauchy-Green strain tensor defined as

2 $${\bar{I}}_{1}={\bar{\lambda }}_{1}^{2}+{\bar{\lambda }}_{2}^{2}+{\bar{\lambda }}_{3}^{2}$$

where the deviatoric stretches assume the form: ${\bar{\lambda }}_{i}={J}^{-\frac{1}{3}}{\lambda }_{i}$ and *J* is the volumetric deformation. The material parameters can then be expressed as, ${C}_{10}={\mu }_{0}/2$ and ${D}_{1}=2/{K}_{0}\,$, where ${\mu }_{0}$ is the initial shear modulus and ${K}_{0}$ is the bulk modulus.

Each thin lamina between the connections of the reinforcing scaffolds was assumed to behave as a linear elastic orthotropic material in plane stress. Due to the material property symmetry, the stress-strain relations expressed in a local coordinate system aligned with the fibers constituting the lamina in the 1-direction, are related by

3 $$[\begin{array}{c}{\varepsilon }_{11}\\ {\varepsilon }_{22}\\ {\varepsilon }_{12}\end{array}]=[\begin{array}{ccc}{S}_{11} & {S}_{12} & 0\\ {S}_{12} & {S}_{22} & 0\\ 0 & 0 & {S}_{66}\end{array}]\,[\begin{array}{c}{\sigma }_{11}\\ {\sigma }_{22}\\ {\sigma }_{12}\end{array}]$$

where the compliance elements S*ij*, are related to the engineering constants as, ${S}_{11}\,=\,\,1/{E}_{1}$, ${S}_{12}=-{\upsilon }_{12}/{E}_{1}$ = $-{\upsilon }_{21}/{E}_{2}$, ${S}_{22}=1/{E}_{2}$, $\,{S}_{66}=1/{G}_{12}$ and ${E}_{i}$, ${\upsilon }_{ij}$ and ${G}_{ij}$ are the elastic stiffness, Poisson’s ratio and shear modulus constants, respectively. The elements $\,{S}_{44}=1/{G}_{23}$, $\,{S}_{55}=1/{G}_{13}$ were also required to fully define the scaffold material properties, since each lamina might undergo transverse shear deformation^27^. The fibre interconnections at the fibre-crossing points were assumed to behave like a column with stiffer transversal properties than that of the lamina. For this reason, an additional stiffness parameter *E*_*int*_ was introduced to account for these interconnected fibres. These interconnections were modelled as linearly elastic. The material behaviour of the total scaffold thus was described by 6 parameters, 5 for the lamina and 1 for the interconnections.

#### Assessment of the constitutive parameters

The unknown parameters of the Neo-Hookean hydrogel model $({C}_{10}$ and ${D}_{1}$), were determined by fitting the reaction force-displacement curves as measured in the experiment (see above) and those predicted from the hyperelastic continuum FE model, implementing an initial reasonable guess for the material parameters. A Matlab script was developed to optimize the material parameters using a multidimensional unconstrained nonlinear minimization method^28^. The objective function used was defined by,

4 $$f=\frac{1}{n}\sum _{i=1}^{n}{(\frac{R{F}_{Exp}^{i}-R{F}_{FEM}^{i}}{R{F}_{Exp}^{i}})}^{2}$$

where $R{F}_{Exp}^{i}$ and $R{F}_{FEM}^{i}$ are the experimental and predicted reaction forces, respectively, and n = 350 is number of data points from the curve considered. The fitting procedure was repeated till the RF resulting from the computational simulations fell within the standard deviation of the experimental results.

For the lamina, the longitudinal stiffness ${E}_{1}$ was assumed to be that of the single fibre tensile stiffness as measured in the experiment divided by a factor two. The latter was done to account for the porosity introduced by the stacking nature of the fibres. The remaining 5 parameters describing the orthotropic elastic behaviour of the lamina (${E}_{2}$, ${\upsilon }_{12}$, ${G}_{12}$, ${G}_{23}$, ${G}_{13}$) as well as the additional stiffness assumed at the interconnections (*E*_*int*_), were determined by fitting the reaction force-displacement curves as measured in the experiment (see above) and those predicted from the linear elastic continuum FE model, representing only the fibre scaffold (without gel) with an 800 µm fibre spacing. The same fitting procedure as used for the hydrogel parameters was used, again starting with an initial reasonable guess for the material parameters.

### Micro FE model

#### High-resolution micro-CT analysis

High-resolution images of the real composite construct architectures were obtained through different stages of compressive deformation with the use of a micro-CT scanner (µCT 80, Scanco Medical AG, Switzerland). Pure scaffolds and reinforced gels with a fibre spacing of 200 and 800 µm were analysed at increasing compressive strain levels of 0, 15, 30 and 45%. The micro-CT device was equipped with a custom-made loading device. This loading system consists of a supporting tube driven by a bolt system and two spacers^23^ (Fig. 2C). When tightened, the system enabled the compression of the constructs to the required deformation level. A water-based contrast agent solution (Ioversol, Optiray 300 ^TM^, Mallinckrodt Pharmaceuticals) was used in the reinforced constructs for staining the GelMA hydrogel. The acquisition parameters were set to a voltage of 70 kVp; an intensity of 114 µA and an integration time of 300 ms. After scanning, a Gauss filter was applied (sigma = 1, support = 0.8 voxel) and images were subsequently segmented. A global threshold of 24 per mile and 105–195 per mile were used for the pure scaffold and scaffold region on the reinforced hydrogel constructs.

#### Micro- FE analysis

Micro-FE models were created directly from the segmented images by using a voxel conversion procedure. Equally sized brick elements were used to generate the FE mesh of the scaffolds (≈3 M elements) and hydrogel composite phase (≈30 M elements). The micro-CT images of each deformed state, *i*.*e*. 0, 15, 30 and 45%, were used to generate the micro-FE model, and a low-friction compression test prescribing a 1% strain was performed on the deformed configuration to investigate the stiffness and the stress distribution in the deformed state. Each material phase, i.e. scaffold (E_fibscaff_, υ_fib scaff_) and hydrogel (E_gel_, υ_gel_), was modelled as a homogenous linear elastic material, described by a Young’s modulus and a Poisson’s ratio. The Poisson’s ratio of the fibre scaffold was set as 0.3, equal to bulk PLC^29^, that of the hydrogel was set to 0.49, representing an almost incompressible material as witness by the lateral strain measurements during the compression test. The Young’s modulus of the hydrogel and of fibre were determined by fitting the reaction force as measured the experiment at 15% strain (see above) and those predicted from the micro-FE at the deformed configuration of 15% strain. Micro-FE analysis and imaging processing were performed using Scanco Finite Element software IPLFE v5.16 (Scanco Medical AG, Switzerland).

## Electronic supplementary material

Supplementary Information
