# Supplementary material for: Mechanical behavior of a soft hydrogel reinforced with three-dimensional printed microfibre scaffolds
Source: Sci Rep. 2018 Jan 19;8:1245. doi: 10.1038/s41598-018-19502-y (PMC5775327; doi:10.1038/s41598-018-19502-y)
Supplement: Supplementary file 1 — Supplementary Information [file 41598_2018_19502_MOESM1_ESM.pdf]

Mechanical behavior of a soft hydrogel reinforced with three-dimensional printed microfibre scaffolds

Miguel Castilho, Gernot Hochleitner, Wouter Wilson, Bert van Rietbergen, Paul D. Dalton, Jürgen Groll, Jos Malda and Keita Ito

Supplementary information

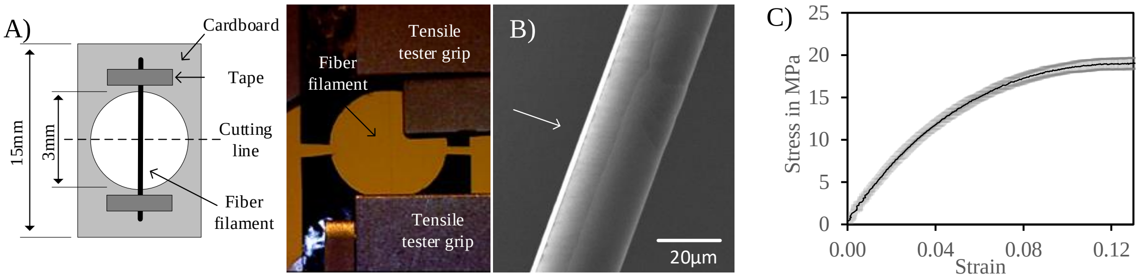

Figure S1. A) Method used for tensile measurement on single PCL melt electrospun fibres. PCL fibres were fixed on a black cardboard and then mounted in the grips of the tensile tester. Before testing, fibre alignment was visually checked and both sides of the cardboard were cut. B) SEM image showing the fiber morphology before testing, with the white arrow indicating the fibre bottom, where it touched the collector plate, evidencing its cylindrical geometry. C) stress- strain curves of single fiber loaded under tension. Error bars represent the experimental standard deviation of a given stress value (n=5).

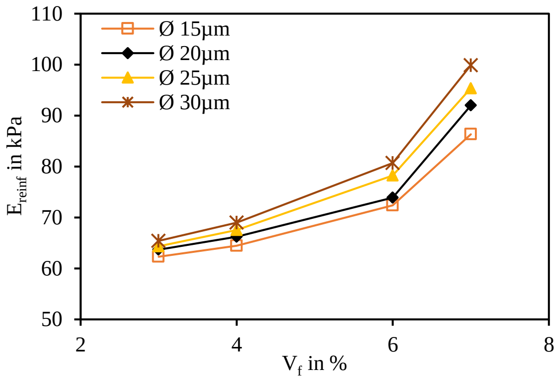

Figure S2. Effect of fiber diameter (same as lamina thickness) on the predicted stiffnesses of the reinforced constructs using continuum FE model.

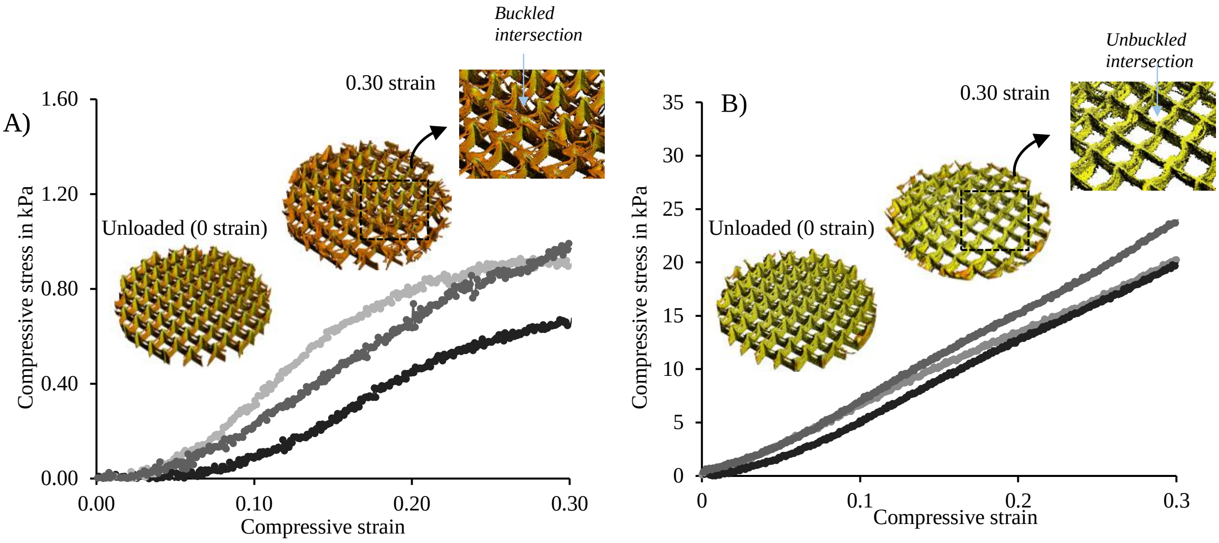

Figure S3. Typical engineering stress–strain curves of A) three individual samples of fibre scaffold with a Vf= 3% and B) three individual samples of reinforced hydrogel with a Vf=3%. The stress-strain curves are related to the respective compressive stress distribution obtained from Micro-FE model at two strain levels, 0 and 30%. Micro-FE analysis was performed by applying a axial compressive strain of 1% at underformed and deformed configurations, which were obtained with the micro-compression device illustrated in Figure 2. Combination of the fibre scaffold with the hydrogel clearly affect the buckling behaviour of the fibre scaffold alone as represented by detail images of a buckled fibre intersection and of the hydrogel limiting its buckling.

**Geometry parameters**		**Mesh characteristics**		
**Part**	**Dimensions in mm**	**Element Type**	**Element Number**	**Nodes**
Fibre scaffold	(h x a x b x t)			
Square boxes	2 x 0.4 x 0.8 x 0.02	M3D4	11088	11250
	2 x 0.3 x 0.6 x 0.02		13200	13275
	2 x 0.2 x 0.4 x 0.02		21736	21420
	2 x 0.1 x 0.2 x 0.02		40480	37035
Interconnections	(h) 2	T3D2	1	2
Hydrogel matrix	(r x h) 2.5 x 2	C3D8H	42780	46368

Table S1. Geometrical parameters and mesh characteristics of the continuum FE model used in this study. Geometric parameters are illustrated in Fig. 2B. The reinforced constructs result from the combination of the different size scaffolds with the hydrogel matrix.
